# Supplementary material for: More than just a bad day? Traumatic life events and self-control in old age
Source: PLoS One. 2023 Feb 8;18(2):e0266312. doi: 10.1371/journal.pone.0266312 (PMC9907851; doi:10.1371/journal.pone.0266312)
Supplement: S1 Table — (DOCX) [file pone.0266312.s001.docx]

**S1 Table**. **Description of variables**

| **Variable** | **Survey questions and coding** |
| --- | --- |
| Trauma variables: |  |
| Physical attack | “Were you the victim of a serious physical attack or assault in your life?” |
| Natural disaster | “Have you even been in a major fire, flood, earthquake, or other natural disaster?” |
| Life-threatening illness | “Did you ever have a life-threatening illness or accident?” |
| Baseline covariates: |  |
| Age | Age at the time of survey |
| Female | Female = 1; male = 0 |
| Black or Hispanic | African American or Hispanic = 1; White or other races = 0 |
| High school graduate | Years of schooling = 12 |
| Some college | Years of schooling = 13, 14, or 15 |
| College and above | Years of schooling >= 16 |
| Married | Married = 1; separated, divorced, widowed, or never married = 0 |
| No. of living children | Number of living children |
| Poor or fair health | Self-rated health: poor or fair = 1; good, very good, or excellent = 0 |
| No. of health insurance | Count of employer-provided / private health insurance plans |
| OOP medical spending | OOP medical expenditures since the last interview |
| Employed | Works full-time or part-time = 1; unemployed or retired = 0 |
| Unemployed | Unemployed = 1; works full-time or part-time or retired = 0 |
| Household income | Sum of household earnings, pensions and annuities, Social Security benefits, government transfers, capital income, and other income |
| Total net worth | Net value of checking and savings accounts, bonds and bond funds, stocks and stock mutual funds, retirement accounts, primary home, real estate, business equity, vehicles, and other savings |
| Early-life characteristics: |  |
| Place of birth | Born in South Atlantic, West South Central, and East South Central = 1; other regions = 0 |
| Mother’s education background | Mother’s years of education |
| Father’s education background | Father’s years of education |
| Quality of relationship with mother | “I had a good relationship with my mother before age 18” (Agree or strongly agree = 1; neither agree nor disagree, disagree, or strongly disagree = 0) |
| Quality of relationship with father | “I had a good relationship with my father before age 18” (Agree or strongly agree = 1; neither agree nor disagree, disagree, or strongly disagree = 0) |
| Substance abuse of parents | “Before you were 18 years old, did either of your parents drink or use drugs so often that it caused problems in the family?” (yes = 1; no = 0) |
| OOP medical spending (earlier wave) | OOP medical expenditures recorded in the 1998 wave (HRS, AHEAD, CODA, and WB cohorts); OOP medical expenditures recorded in the 2004 wave (EBB cohort) |
| Employed (earlier wave) | Employed in 1998 (HRS, AHEAD, CODA, and WB cohorts); employed in 2004 (EBB cohort) |
| Unemployed (earlier wave) | Unemployed in 1998 (HRS, AHEAD, CODA, and WB cohorts); unemployed in 2004 (EBB cohort) |
| Household income (earlier wave) | Household income in 1998 (HRS, AHEAD, CODA, and WB cohorts); household income in 2004 (EBB cohort) |
| Total net worth (earlier wave) | Total net worth in 1998 (HRS, AHEAD, CODA, and WB cohorts); total net worth in 2004 (EBB cohort) |
