# Supplementary material for: More than just a bad day? Traumatic life events and self-control in old age
Source: PLoS One. 2023 Feb 8;18(2):e0266312. doi: 10.1371/journal.pone.0266312 (PMC9907851; doi:10.1371/journal.pone.0266312)
Supplement: Attachment — Submitted filename: Reviewer2.pdf [file pone.0266312.s002.pdf]

The paper investigates whether exposure to traumatic events shapes self-control at older ages. Using data from the US Health and Retirement Study, the authors find that experiencing serious physical attacks or assaults is associated with a significant reduction in self-control. Moreover, traumatic experiences in early life have a long-lasting effect on self-control.

The paper is well written, and the research question is clearly stated. Still, I have concerns about several limitations of the analysis.

- Representativeness of the sample. In section 3.1, the authors provide information about the sample selection and the composition of the estimating sample. Though, they say nothing about the response rates to the relevant questions and do not discuss how much the estimating sample represents Americans aged 50+.

- Measure of self-control. The discussion of the measure of self-control is poor, and its distribution is not adequately discussed. The features of the outcome measure are crucial to design the empirical strategy. Self-control is measured on a six-point (ordered and aggregated) scale, and it is seemingly treated as a continuous variable, but no discussion on that is provided.

- Empirical strategy. Related to the previous point, the authors estimate the model by a linear OLS and do not provide any robustness check with alternative estimators. Why not estimate the model by nonlinear estimators for ordered outcomes?

- Non-random exposure to a traumatic experience. (Luckily) only 7% of the respondents have experienced a physical assault, which is a rare event. On the other hand, almost 40% of the sample witnessed some trauma. The types of trauma the authors consider are intrinsically different the one from the other, and the regression outcomes somehow confirm that. Especially when using propensity score matching, the traumatic events should be distinguished as the determinants of the probability of experiencing each of them are highly heterogeneous.

- Discussion of the results. It is surprising how much the results are sensitive to including early life controls and personality traits in terms of magnitude and statistical significance of the estimated effects. This should warn about endogeneity issues for some of the covariates, omitted variable problems, and non-random exposure to trauma. I do not see any discussion on this evidence.

- Limitations of the study. The authors acknowledge some limitations of their analysis, which are, in my opinion, more serious than what was stated. I think these drawbacks strongly affect the identification strategy and definitely prevent from estimating a causal relationship between exposure to trauma and self-control.

- Policy implications. The policy implications discussed in the conclusions are very weak. I have the impression the authors stretch their reasoning too much simply not to leave some policy implication out of the picture.
