# Supplementary material for: More than just a bad day? Traumatic life events and self-control in old age
Source: PLoS One. 2023 Feb 8;18(2):e0266312. doi: 10.1371/journal.pone.0266312 (PMC9907851; doi:10.1371/journal.pone.0266312)
Supplement: Attachment — Submitted filename: Response to Reviewers.docx [file pone.0266312.s003.docx]

Dear editor and referees,

We wish to thank all of you for careful reading of the paper and valuable comments. We have paid close attention to your concerns and have addressed them in the revised manuscript thoughtfully and, hopefully, to your satisfaction. Below you will find detailed responses to concerns raised in your report. Please also see our responses to the other referee’s comments to understand why and how we revised other parts of the manuscript.

Reviewer #1

Comment R1-1.

*It is suggested to include in the title the times of occurrence of the traumatic experiences, early, late or in old age, so that the reader understands a little more what the research is about without having to read the full text, as well as in the introduction , since those are the variables that are being evaluated mainly in the research*

Response R1-1.

I agree with the reviewer that more information on the occurrence of the traumatic event would help authors better grasp of the topic. However, in this study the time that the traumatic event occurred vary across individuals’ lifetime; some occurred in childhood, and others occurred in middle age. To reflect this study design, we have updated the title with “More than just a bad day? Traumatic life events and self-control in old age”. The main text of the manuscript is also updated accordingly; we consistently used the term “traumatic life events” instead of “traumatic experiences”.

Comment R1-2.

*It is requested to improve figure 1, it does not have good quality, as well as to identify or rather to differentiate the axes, especially to identify the one that corresponds to age to avoid confusion, especially for new researchers in the area, who wish to continue their research.*

Response R1-2.

Agree; we have removed y-label and clarified the unit of the horizontal axis by adding “years elapsed” within the parenthesis. The revised figure displays the overall distribution of “the years elapsed since the event” along with its mean and boundaries of standard deviation.

Reviewer #2

Comment R2-1.

*Representativeness of the sample. In section 3.1, the authors provide information about the sample selection and the composition of the estimating sample. Though, they say nothing about the response rates to the relevant questions and do not discuss how much the estimating sample represents Americans aged 50+.*

Response R2-1.

There were 13,101 respondents aged 50 to 80 who have responded to the Leave-Behind (LB) module in 2010 and 2012. After dropping observations with missing values, a total of 8767 observations were retained in the analytic sample.

| Sample description | Number of observations |
| --- | --- |
| Participants aged 50 to 80 who have responded to the LB module in 2010 and 2012 | 13,101 |
| Participants aged 50 to 80 who have measurements of self-control and traumatic life events | 12,273 |
| Excluding missing values in early life controls | 10,003 |
| Excluding missing values in labor supply, household income, total net worth, and medical expenses in the 1998 and 2004 waves | 8936 |
| Excluding missing values in demographic and socioeconomic variables | 8767 |

Below is the average sample characteristics before and after dropping missing values:

|  | N=12,273 | N=8767 |
| --- | --- | --- |
| Self-control | 4.35 | 4.36 |
| Physical attack | 0.08 | 0.07 |
| Natural disaster | 0.18 | 0.17 |
| Life-threatening illness | 0.25 | 0.25 |

The two sample t-test results showed no significant differences in self-control and traumatic life events (at the 5% level) across the two samples. Thus, it is quite unlikely that the sample selection process confounds our estimates of the association between traumatic life events and self-control.

In 2010 and 2012, the response rates for the LB module were 73.1% and 72.7%, respectively. Our results and estimates were weighted by the sample weights that reflect the complex sampling procedures of the HRS and LB module. Please note that, throughout the manuscript we have avoided making a claim about the representativeness of our analytic sample. While we expect that our sample is plausibly similar to Americans aged 50 to 80, losing about one-third of the LB module data makes us unable to put forth a definitive claim about representativeness. In the revised manuscript, we updated the data description section with the response rates of the LB module and weighting of the results using sample weights (page 8).

Comment R2-2.

*Measure of self-control. The discussion of the measure of self-control is poor, and its distribution is not adequately discussed. The features of the outcome measure are crucial to design the empirical strategy. Self-control is measured on a six-point (ordered and aggregated) scale, and it is seemingly treated as a continuous variable, but no discussion on that is provided.*

Response R2-2.

The subsection for self-control is further expanded with additional explanation of the measure and its validity. Please note that self-control is measured on a continuous scale of 1 to 6, with higher score representing greater self-control. Below is the distribution of the self-control score:

Comment R2-3.

*Empirical strategy. Related to the previous point, the authors estimate the model by a linear OLS and do not provide any robustness check with alternative estimators. Why not estimate the model by nonlinear estimators for ordered outcomes?*

Response R2-3.

As discussed above, the self-control score is a continuous measure, and hence we believe linear OLS with two-way clustered standard error is the ideal specification. We transformed the self-control score using a log function and re-estimated the models, but the inferences remain unchanged.

Comment R2-4.

*Non-random exposure to a traumatic experience. (Luckily) only 7% of the respondents have experienced a physical assault, which is a rare event. On the other hand, almost 40% of the sample witnessed some trauma. The types of trauma the authors consider are intrinsically different the one from the other, and the regression outcomes somehow confirm that. Especially when using propensity score matching, the traumatic events should be distinguished as the determinants of the probability of experiencing each of them are highly heterogeneous.*

Response R2-4.

Agree; we updated the regressions for propensity score matching as suggested. The propensity score is estimated by matching the sample by attack, disaster, and illness, respectively. Each regression for matching controls for a full set of covariates including other traumatic events. The weighted regression results are presented in columns 5-7 in Table 2 (page 14). Overall, physical attack remains significantly related to self-control at the 5% level.

Comment R2-5.

*Discussion of the results. It is surprising how much the results are sensitive to including early life controls and personality traits in terms of magnitude and statistical significance of the estimated effects. This should warn about endogeneity issues for some of the covariates, omitted variable problems, and non-random exposure to trauma. I do not see any discussion on this evidence.*

*Limitations of the study. The authors acknowledge some limitations of their analysis, which are, in my opinion, more serious than what was stated. I think these drawbacks strongly affect the identification strategy and definitely prevent from estimating a causal relationship between exposure to trauma and self-control.*

Response R2-5.

We updated our discussion of limitations accordingly (page 17). Please note that this study does not give causal inference regarding the effect of traumatic life events. We share the concern with the reviewer, but this limitation is not avoidable unless we use a randomized controlled trial (RCT). While the RCT might be a golden rule for causal inference, it is also limited in that it gives the short-term, immediate impact of the treatment and lacks generalizability. This study, though limited in some regards, has the benefits of examining the potential long-term association between traumatic life events and self-control. We believe this study makes a useful contribution to the literature on how negative life events alter economic preferences and behaviors.

Comment R2-7.

*Policy implications. The policy implications discussed in the conclusions are very weak. I have the impression the authors stretch their reasoning too much simply not to leave some policy implication out of the picture.*

Response R2-7.

Our discussion of implication is re-framed around lifetime consequences of traumatic life events. We strived to ensure that our discussion is based on our findings, and not going beyond the scope of this study. Please check the last paragraph on page 18.
