# Supplementary material for: More than just a bad day? Traumatic life events and self-control in old age
Source: PLoS One. 2023 Feb 8;18(2):e0266312. doi: 10.1371/journal.pone.0266312 (PMC9907851; doi:10.1371/journal.pone.0266312)
Supplement: Attachment — Submitted filename: response to reviewers.docx [file pone.0266312.s004.docx]

Reviewer #2

Comment R2-1.

*At the conceptual level, a range of constructs all assessed in the HRS could be good candidate as dependent variables such as depressed mood, well-being, or attitudes to aging. I found the rationale behind choosing self-control not sufficiently elaborated. This certainly is a key construct in early life or as a ‘life starting’ variable as the Moffitt et al study has convincingly shown. However, I would argue that the construct becomes less important for older adults compared for example with depression. Two request: (1) Please provide a more convincing argument why you chose self-control; (2) what is the difference between self-control and locus of control, which eventually has also been assessed in the HRS?*

Response R2-1.

Several studies in Economics and Business showed that self-control is associated with financial behavior and well-being in adulthood. There also exists research (based on the HRS data) that showed that self-control problem is associated with asset decumulation and financial distress at older ages. Most studies in Economics examine adult sample to study self-control problems because economic behavior is not observable in children and adolescents. We have restructured the first paragraph of Introduction to clarify why self-control is important for adults and older adults. We appreciate your suggestion to consider "depressed mood, well-being, or attitudes to aging", but we feel these outcomes are beyond the scope of this study.

As far as we understand, locus of control is context-specific, while self-control is more of the general concept, measuring one's overall self-regulation ability. We see that the HRS offers a number of questions measuring domain-specific self-efficacy, such as health, social life, and finances. Examining locus of control would be an interesting avenue for future research, but it may not be the main research idea for this study.

Comment R2-2.

*Are you trying to make an argument that early traumatic experiences are also initiate structural or physiological brain impairment that may last for the next decades? If so, please offer a more convincing argument, ideally backed by brain-imaging data. If so, I would expect that cognitive functioning might indeed be a better dependent variable than self-control.*

Response R2-2.

The last paragraph of Section 2.1 offers a brief review of the literature on trauma exposure and brain development. This section is further expanded with references on brain scanning research.

Regarding the second point, we believe the link between traumatic experience and cognitive loss operates through other mechanisms, mainly depressive symptoms. One of the triggers for cognitive loss at older ages, as reviewer indicated in other comments, is childhood adversities. We think this is a different research direction because childhood adversities are not necessarily traumatic, and not all traumatic events lead to depressive symptoms. The experiences of a traumatic event may or may not trigger depression, and cognitive loss may or may not occur depending on whether traumatic events impair mental health. In this study, we are talking about life events that could be potentially traumatic, such as physical assault, natural disaster, and life-threatening illness. People may feel mental shock and suffer from trauma when they experience, for instance, the threat of homicide or a large-scale tsunami. However, these events do not necessarily incur depressive symptoms. Examining cognitive loss for older adults sounds potentially promising, but it is not the main focus of this study.

Comment R2-3.

*I am confused that on p. 3 in the intro part already the findings of the study are described? This is at least totally unusual in my ‘journal world’.*

Response R2-3.

We believe it is not unusual in Economics and Business to give a brief summary of findings in Introduction. As this paper intends to appeal to behavioral economists, we prefer to follow their practice.

Comment R2-4.

*Referring to early trauma experiences cannot in my view not be separated from childhood adversities. To my knowledge, linkages among childhood adversities and late-life adverse outcomes have already been researched with the HRS data. Are you indeed heading for the full picture of traumatic experiences, i.e., childhood adversities and what happens later until base line assessment? Or even during the different waves of the HRS running since something like 1993? This needs to crystal-clear in my view.*

Response R2-4.

Our measure of traumatic life events includes traumatic events experienced in early childhood and those experienced as adults. This is why we defined a critical age of brain maturity and split the effects of traumatic life events according to this threshold (Table 3). Our main finding was that there was no difference between a physical attack experienced before the age of brain maturity and the attack experienced after that, in terms of its effect on self-control.

To our knowledge, prior research on "childhood adversities" examined other domains of adversities such as economic insecurity, parental job loss, divorce of parents, loss of siblings, etc. These events might be the source of stressful experiences, but they might not create "traumatic" experiences like the ones considered in this study. We believe their effect, if any, operates through the socioeconomic channels over the course of life, but is unlikely through the neurophysiological changes. Ours is not related to this "childhood adversity" literature because the expected effect of events operates through a different mechanism.

Comment R2-5.

*On p. 4 you mention self-control and “associated well-being outcomes”. I agree that this would be important, but which such well-outcomes were included in your study? Does this also mean that you maintain that self-control is a mediator for developmental outcomes? If so, then this needs to be shown empirically.*

Response R2-5.

With this description, we are emphasizing the importance of self-control in adulthood and for older ages. This description does not mean that this study is examining the well-being consequences of self-control. We believe our main topic (association between traumatic life events and self-control) and its contribution to the literature was clearly explained in Introduction. Please notice that, in response to the first comment we introduced studies on the effects of self-control on economic well-being for adults and older adults (first paragraph of Introduction) and clarified why we are studying this issue.

Comment R2-6.

*It seems that the full flow of argument is driven by a ‘risk perspective’. However, findings on resilience and adaptational potentials seems to be ignored to a large extent. For example, we know that long-term development comes with habituation and adaptation. Therefore, at least the kind of traumatic events need to be differentiated in those being of very high risk and those of medium or low risk for the rest of lifespan development.*

Response R2-6.

We agree that there could be habituation and adaptation after experiencing traumatic events. However, we are not able to examine such mechanisms because the HRS data do not offer measurements of what has happened over the course of life. Moreover, we believe this topic goes beyond the topic of this study. We would like to maintain a tight focus on the association between traumatic life events and self-control for older adults and empirically validate this claim. This issue might be an interesting avenue for future research, and we leave it to other scholars.

Comment R2-7.

*There is also talk about traumatic experiences and economic preferences. Is this part of the research aims of the study?*

Response R2-7.

Yes, as discussed at the outset of this manuscript, this study intends to appeal to a subset of behavioral economists who claim that trauma exposure has a lasting influence on economic preferences.

Comment R2-8.

*Just to make sure: Did you control for self-control level in previous waves or at baseline in your statistical model? Sorry, for having missed this.*

Response R2-8.

The sampled respondents answered self-control questions only once during the study periods. Therefore, self-control in the previous wave was not available in our data.

Even if they were measured in data, they should not be included in regressions because traumatic life events in year t-1 (or baseline) would absorb all the effects of traumatic life events experienced earlier, and leaves only between-wave differences in self-control to be explained by traumatic life events of the past. This is not what we intend to estimate. Our research question corresponds to the lasting influence of traumatic life events. This can be best addressed by regression of current self-control on lifetime exposure to traumatic events and its potential confounders.

Comment R2-9.

*As a life-span and aging researcher, I see a clear need to differentiate for different life phases in late-life. Controlling for age is not sufficient. For example, there is evidence that early trauma may gain a new momentum in advanced old age, because over vulnerability may impair psychological protection.*

Response R2-9.

To respond to this comment, we have estimated the main regression separately for respondents aged 50 to 60, 61 to 70, and 71 to 80. We found that the coefficient estimate on physical attack was -0.129*** for participants aged 50-60, -0.169** for ages 61-70, and -0.129** for ages 71-80, where *** and ** indicate statistical significance at the 1% and 5% level, respectively. Here, we confirm that the association between physical attack and self-control (i.e., main finding) is robust to the age of respondent.
